# Zebrafish: a novel research tool for cardiac (patho)electrophysiology and ion channel disorders

**DOI:** 10.3389/fphys.2012.00255

**Published:** 2012-07-10

**Authors:** Arie O. Verkerk, Carol Ann Remme

**Affiliations:** ^1^Department of Anatomy, Embryology, and Physiology, Academic Medical Center, University of AmsterdamAmsterdam, Netherlands; ^2^Department of Experimental Cardiology, Academic Medical Center, University of AmsterdamAmsterdam, Netherlands

**Keywords:** action potential, arrhythmia, cardiac electrophysiology, ion channel, ion channelopathy, patch-clamp, zebrafish

## Abstract

The zebrafish is a cold-blooded tropical freshwater teleost with two-chamber heart morphology. A major advantage of the zebrafish for heart studies is that the embryo is transparent, allowing for easy assessment of heart development, heart rate analysis and phenotypic characterization. Moreover, rapid and effective gene-specific knockdown can be achieved using morpholino oligonucleotides. Lastly, zebrafish are small in size, are easy to maintain and house, grow fast, and have large offspring size, making them a cost-efficient research model. Zebrafish embryonic and adult heart rates as well as action potential (AP) shape and duration and electrocardiogram morphology closely resemble those of humans. However, whether the zebrafish is truly an attractive alternative model for human cardiac electrophysiology depends on the presence and gating properties of the various ion channels in the zebrafish heart, but studies into the latter are as yet limited. The rapid component of the delayed rectifier K^+^ current (I_Kr_) remains the best characterized and validated ion current in zebrafish myocytes, and zebrafish may represent a valuable model to investigate human I_Kr_ channel-related disease, including long QT syndrome. Arguments against the use of zebrafish as model for human cardiac (patho)electrophysiology include its cold-bloodedness and two-chamber heart morphology, absence of t-tubuli, sarcoplamatic reticulum function, and a different profile of various depolarizing and repolarizing ion channels, including a limited Na^+^ current density. Based on the currently available literature, we propose that zebrafish may constitute a relevant research model for investigating ion channel disorders associated with abnormal repolarization, but may be less suitable for studying depolarization disorders or Ca^2+^-modulated arrhythmias.

## Introduction

To date, genetically modified mice have been predominantly used to investigate and model human cardiac diseases, including patho-electrophysiological conditions. Although mouse models have provided valuable insight into the role of many ion channels in healthy and diseased state, they also have limitations due to their intrinsic basal high heart rate and extremely fast and large phase-1 repolarization which results in a short action potential (AP) with a very negative plateau phase potential. Furthermore, *in vivo* investigation of mouse models often requires invasive imaging and monitoring techniques. Finally, generation and maintenance of mouse lines is time-consuming and expensive.

In the last decade, the zebrafish (*Danio rerio*), a tropical freshwater teleost, has been increasingly used for various human-related disease studies (Beis and Stainier, 2006; Williams, 2010). Despite its cold-bloodedness and two-chamber heart morphology, the zebrafish has been suggested as a useful model for studies of human heart development and cardiac (patho)electrophysiology. A major advantage of the zebrafish for heart studies is it that the embryo is transparent, allowing for easy assessment of heart development, heart rate analysis and phenotypic characterization by direct visual inspection (Baker et al., 1997; Bakkers, 2011). Furthermore, using optogenetics combined with transgenic expression of light-gated ion channels in zebrafish hearts, cardiac pacemaker cells can be located and quickly and reversibly activated and deactivated in various sub-compartments of the cardiac conduction system, enabling investigations into the effects of disturbed heart rhythms on cardiac performance (Arrenberg et al., 2010). An additional advantage is that an intact blood circulation is not required for proper function of fish embryos and hearts, since diffusion of nutrients is sufficient for their survival. Therefore, *in vivo* and *ex vivo* functional studies can be performed easily without complications due to nutritional deficiency or secondary deterioration (Baker et al., 1997; Peal et al., 2011). Moreover, rapid and effective gene-specific antisense knockdown using morpholino oligonucleotides allows for relatively quick *in vivo* functional characterization of the activity and function of genes of interest (Bedell et al., 2011). Lastly, zebrafish are small in size, are easy to maintain and house, grow fast, and have large offspring size, making them a cost-efficient research model.

Today, zebrafish whole heart electrical activity is routinely recorded using *in vivo* electrocardiography (ECG) (Leong et al., 2010). In addition, various non-invasive microscopic video analysis methods have been developed to determine heart rate (Chan et al., 2009; Yoshida et al., 2009), to quantify ventricular fractional shortening [a measure of systolic contractile function (Denvir et al., 2008; Fink et al., 2009)], and to analyse blood flow dynamics by tracking movement of erythrocytes or fluorescent molecules introduced into the circulation (Schwerte and Pelster, 2000; Hove et al., 2003). For assessment of cardiac conduction and excitability, Ca^2+^-sensitive fluorescent dyes (Ebert et al., 2005; Langenbacher et al., 2005; Milan et al., 2006) or a fluorescent Ca^2+^indicator transgene [Tg(cmlc2:gCaMP)] (Chi et al., 2008) can be used, and transmembrane APs may be evaluated using voltage-sensitive dyes (Panáková et al., 2010). Application of these voltage-sensitive dyes during so-called optical mapping may also enable detailed investigation of cardiac conduction velocity, activation patterns, and arrhythmias. These high resolution imaging techniques are powerful tools for the study of zebrafish physiology (Jou et al., 2010), but these methods require the complete absence of cardiac contraction. Jou and colleagues (Jou et al., 2010) found that the excitation-contraction uncoupler blebbistatin, but not butanedione monoxime (BDM), abolished contractility without significantly altering AP morphology or of spontaneous APs generation.

The zebrafish as model for studies into human heart development, heart regeneration, and human cardiomyopathy diseases has recently been reviewed in detail (Poss, 2007; Bakkers, 2011). The embryonic and adult zebrafish heart is proposed as an efficient platform for testing of drugs with potential electrophysiological effects on cardiomyocytes (Mittelstadt et al., 2008; Tsai et al., 2011) and for investigating human ion channel function in healthy and diseased state (Milan and Macrae, 2008). However, whether the zebrafish is truly an attractive alternative model for human cardiac electrophysiology depends on the presence and gating properties of the various ion channels in the zebrafish heart. Here we review the available data and literature addressing the suitability of the zebrafish as a research model for human cardiac electrophysiology.

## ECG parameters in zebrafish

**Figure 1:**
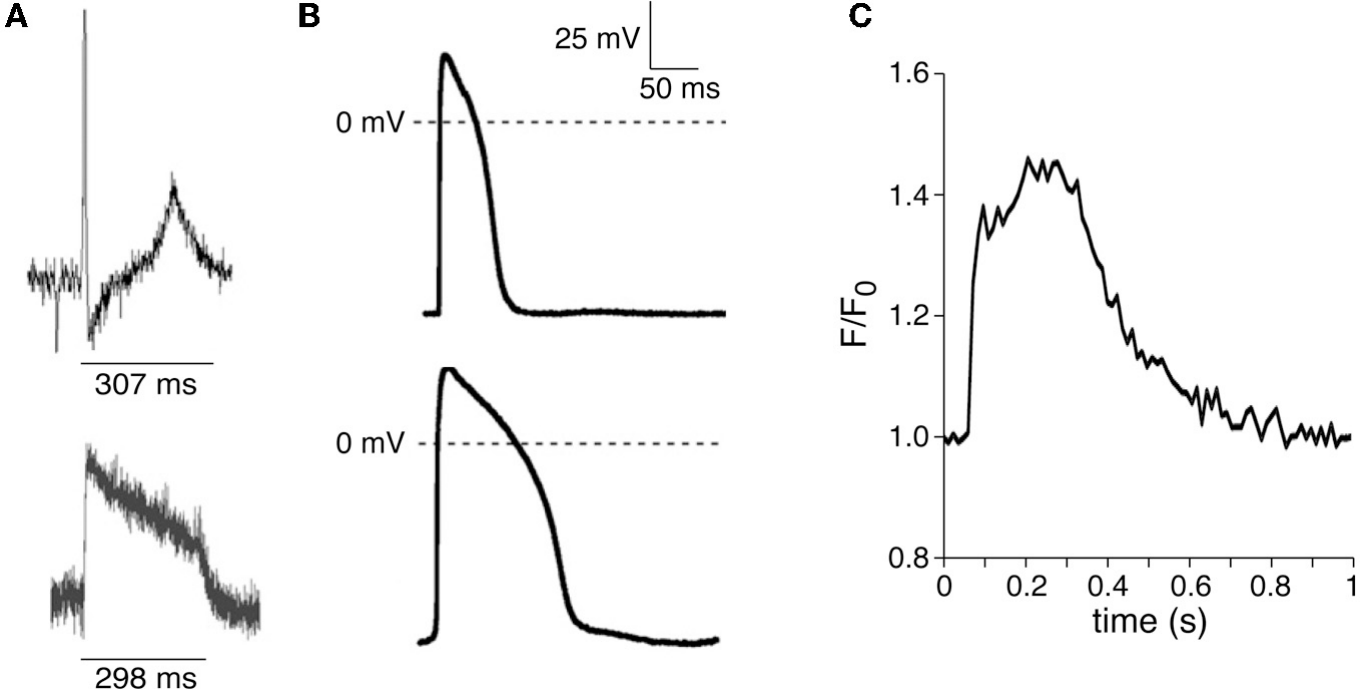
**(A)** Typical ECG recording (*top*) and optically mapped action potential (*bottom*) in adult zebrafish heart. Adapted from Tsai et al. (2011), with the permission of Elsevier. **(B)** Typical atrial (*top*) and ventricular (*bottom*) action potentials recorded from spontaneously beating intact zebrafish hearts. Adapted from Nemtsas et al. (2010), with the permission of Elsevier. **(C)** Typical intracellular Ca^2+^ transient measured within a Fluo-4-loaded isolated ventricular myocyte. Adapted from Zhang et al. (2011), with the permission of the American Physiological Society.

The zebrafish heart is a tubular structure with a single atrium and ventricle. Despite its two-chambered heart morphology, the ECG of the zebrafish is very similar to that of human. Basal heart rate of adult zebrafish is close to that of humans, with a frequency of 120–130 beats/min at 28°C (Barrionuevo and Burggren, 1999; Nemtsas et al., 2010; Tsai et al., 2011), which is the optimal water temperature for this tropical freshwater fish. The heart rate decreases at 25°C and increases at 31°C (Barrionuevo and Burggren, 1999). Pacemaker activity in the zebrafish heart starts in the sinoatrial node located at the sinus venosus (Sedmera et al., 2003). The AP propagates uniformly though the atria with average atrial activation times of ≈20 ms. After a delay of ≈50 ms due to slow propagation in the atrioventricular canal, the ventricle becomes activated first in the apical region. This similarity in propagation of the AP in zebrafish and human hearts mirrors the similarity of ECG morphology (Leong et al., 2010). Like human, zebrafish show a distinct P-wave, QRS-complex, and T-wave on ECG recording (Milan et al., 2006), suggesting that depolarization and repolarization in the zebrafish heart is comparable to that in humans (Leong et al., 2010). The mean QT interval and optically mapped ventricular AP duration is ≈300 ms as shown in Figure 1A (adapted from Tsai et al., 2011). Thus, the QTc interval is slightly shorter than that in humans. However, it must be noted that zebrafish ECG and AP values are obtained at lower temperatures as compared to human, and that human atrial and ventricular APs significantly prolong at lower temperatures (Amos et al., 1996).

## Action potentials

Cardiac APs of zebrafish may be recorded from the intact heart through use of micro-electrodes (Nemtsas et al., 2010), patch-clamp technology (Jou et al., 2010), and voltage-sensitive dyes (Panáková et al., 2010; Wythe et al., 2011). Nemtsas and colleagues (Nemtsas et al., 2010) recorded both atrial and ventricular monophasic APs from intact adult zebrafish hearts that were beating spontaneously at the physiological temperature of 28°C (see Figure 1B for typical examples). They observed a rapid AP upstroke in zebrafish myocardium, but the maximum AP upstroke velocity was substantially lower in zebrafish atria and ventricle than in human and mouse myocardium. The resting membrane potential was similar in zebrafish and human, indicating that the differences in upstroke velocity between species were not due to differences in Na^+^ channel availability. In zebrafish, the AP upstroke was followed by a long-lasting plateau phase that was shorter in atrial than in ventricular tissue, and ended with a phase of rapid terminal repolarization. APs from excised hearts from 48 hours-old zebrafish larvaes displayed similar long-lasting plateau phases, with shorter atrial APs compared to ventricular (Jou et al., 2010; Wythe et al., 2011). In human, atrial and ventricular AP also have a prominent plateau phase with a shorter AP in atria (Koumi et al., 1995; Amos et al., 1996). Nemtsas et al. (2010) concluded that the overall shape of the adult ventricular zebrafish AP is comparable to that of the human heart, and that human APs appear more similar to zebrafish APs than mouse APs. It must be kept in mind, however, that in large mammals (such as human) AP morphology is heterogeneous within both atria and ventricle, and between left and right sides of the heart (see Beuckelmann et al., 1993; Wang et al., 1993; Bénardeau et al., 1996; Näbauer et al., 1996; Gong et al., 2008; Verkerk et al., 2009b; Verkerk et al., and primary references cited therein).

**Figure 2:**
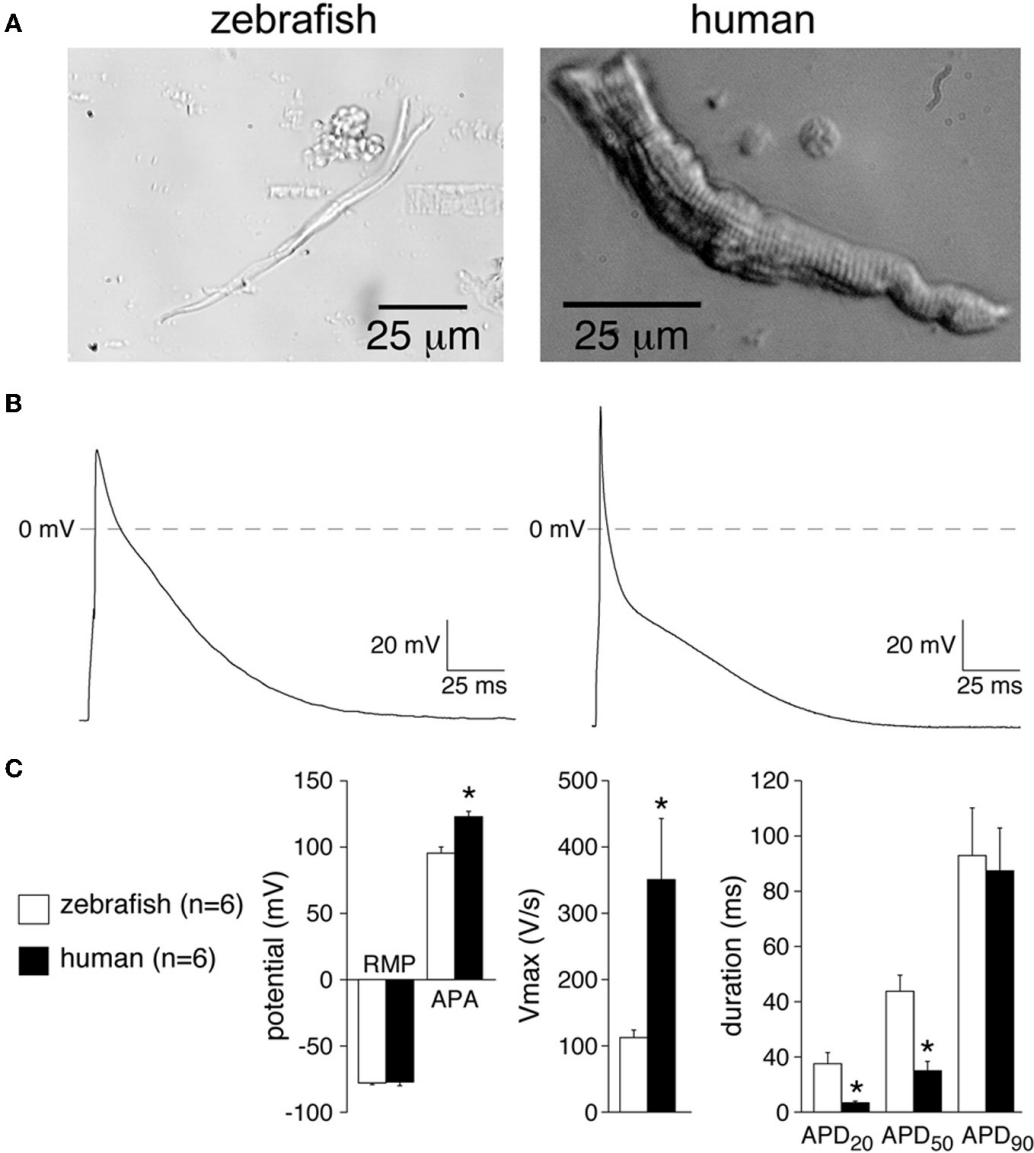
**(A)** Photographs of atrial myocytes of zebrafish and human enzymatically isolated as described in detail previously (Verkerk et al., 2009a), except that the isolation temperature in the zebrafish procedure was decreased to 28°C. **(B)** Typical action potentials (APs) of a single zebrafish and human atrial myocyte measured at 1 Hz. Zebrafish and human atrial APs were recorded at 28 and 36°C, respectively [See Verkerk et al., 2009a (zebrafish) and Verkerk et al., 2007 (human) for solutions used]. **(C)** Average AP characteristics of zebrafish and human atrial myocyte recorded at 1 Hz. RMP, resting membrane potential; APA, AP amplitude; Vmax, maximal AP upstroke velocity; APD_20_, APD_50_, and APD_90_, AP duration at 20, 50, and 90% repolarization, respectively. Values are mean ± SEM; ^*^P < 0.05 (*t*-test).

For recording of cardiac APs in isolated cardiomyocytes, Ca^2+^ tolerant cells need to be isolated through enzymatic dissociation (Brette et al., 2008; Nemtsas et al., 2010; Zhang et al., 2011). Isolated ventricular (Brette et al., 2008) and atrial (Figure 2A) zebrafish myocytes appear rod-shaped, but it is evident from Figure 2A that the zebrafish myocyte is quite narrow compared to that of the human myocyte. It has previously been estimated that freshly isolated ventricular zebrafish myocytes are ≈100 by ≈5 by ≈6 μm in size (length × width × height; Brette et al., 2008). These morphological characteristics of zebrafish myocytes are in contrast with findings in human, where myocytes are much wider. The smaller size of zebrafish myocytes is also evident from their much smaller membrane capacitance. Zebrafish atrial and ventricular myocyte capacitance is ≈26 and ≈30 pF, respectively (Nemtsas et al., 2010), while that of human is ≈90–150 pF (Amos et al., 1996; Verkerk et al., 2007) and ≈165–285 pF (Amos et al., 1996; Li et al., 1998), respectively. It is likely that due to the more narrow shape of the zebrafish myocyte, the relative amount of intercalated disc area is also lower. In mammalian myocytes, intercalated discs, important for AP propagation, are not only found at the cell ends, but also along the lateral sides of the myocyte (Peters et al., 1993). The narrow shape of the zebrafish myocytes may thus influence impulse propagation importantly, but further studies are needed to address this topic in detail.

By patch-clamp analysis, it has been demonstrated that freshly isolated atrial (Figures 2B,C) and ventricular (Brette et al., 2008) myocytes of adult zebrafish display APs with a clear plateau phase. Figure 2B shows typical atrial APs of a zebrafish and human myocyte recorded at 1 Hz; average AP characteristics are summarized in Figure 2C. Compared to human, zebrafish myocytes display a slower AP upstroke velocity resulting in a lower AP amplitude. Zebrafish AP duration during the early phases of repolarization appear longer than in human, but the AP durations at 90% repolarization (APD_90_) are similar (Figure 2C). In isolated ventricular myocytes of adult zebrafish, APD_90_ is ≈150 ms at 0.1 Hz, and it decreases at higher stimulus frequencies. While the frequency dependency is similar to findings in isolated human atrial and ventricular myocytes (Le Grand et al., 1994; Li et al., 2004), the AP duration in single ventricular zebrafish myocytes is much shorter than in isolated human ventricular myocytes (O'Hara et al., 2011).

Thus, embryonic and adult ventricular myocytes of zebrafish show APs with a clear plateau phase and an AP configuration closely resembling that of ventricular myocytes of large mammals, notably human. However, not the AP configuration itself, but the underlying membrane currents will determine whether the zebrafish is suitable as a model for human cardiac electrophysiology.

## Membrane currents

Using patch-clamp analysis and specific ion channel blockers, the presence and function of various inward and outwardly directed membrane currents have previously been investigated in zebrafish cardiomyocytes.

### Na^+^ current

Two orthologs of the cardiac Na^+^ channel have been identified in zebrafish (*scn5Laa* and *scn5Lab*), which both encode and form typical voltage-gated Na^+^ channels/currents (Novak et al., 2006; Chopra et al., 2010). Furthermore, a Na^+^ current (I_Na_) has been observed in both cultured embryonic and freshly isolated adult zebrafish myocytes (Baker et al., 1997; Warren et al., 2001). In single adult atrial myocytes, I_Na_ has a more negative voltage-dependency of inactivation as compared to single adult ventricular myocytes (Warren et al., 2001). Similarly, I_Na_ displays distinct biophysical properties in atrial versus ventricular myocytes in mammalians, with again a more negative voltage-dependency of inactivation (Burashnikov et al., 2007). According to studies by Warren and colleagues (Warren et al., 2001), zebrafish cardiomyocyte I_Na_ density may be up to 4-fold smaller than in mammalian cardiac myocytes, which likely explains the slower AP upstroke velocity found in zebrafish (Nemtsas et al., 2010). Consistent with the importance of I_Na_ in determining AP upstroke (Berecki et al., 2010; and primary refs cited therein), the I_Na_ blocker tetrodotoxin (100 nM) substantially reduced both atrial and ventricular AP upstroke velocity in intact adult hearts (Nemtsas et al., 2010). In contrast, AP duration was not affected by tetrodotoxin, suggesting that a sustained (non-inactivating) I_Na_ is not present under normal conditions. Thus, although similarities with human I_Na_ exists, the limited I_Na_ density and consequent slow AP upstroke velocity in zebrafish may render it less suitable as research model for depolarization disorders.

### Ca^2+^ currents

It has been demonstrated that cultured embryonic and adult freshly isolated zebrafish myocytes display both the T-type and L-type Ca^2+^ current (I_Ca,T_ and I_Ca,L_, respectively) (Baker et al., 1997; Nemtsas et al., 2010). In isolated atrial and ventricular myocytes of adult zebrafish, I_Ca,L_ showed a typical bell-shaped current-voltage (I-V) relationship with a maximum around 0 mV (Brette et al., 2008; Nemtsas et al., 2010; Zhang et al., 2011). In adult zebrafish, the I_Ca,L_ blocker nifedipine significantly shortened the plateau phase and consequently the AP duration in both atria and ventricles (Nemtsas et al., 2010). The I_Ca,L_ activator BayK8644 prolonged QTc interval in a dose-dependent manner (Tsai et al., 2011). These experiments demonstrate that the requirement of I_Ca,L_ for shaping AP duration is conserved between zebrafish and mammals (Nemtsas et al., 2010). The presence of I_Ca,T_ in zebrafish myocytes contrasts with findings in adult human working myocardium (Ono and Iijima, 2005). The effects of I_Ca,T_ blockers on zebrafish AP configuration has not yet been investigated, and the functional relevance of I_Ca,T_ in zebrafish myocytes thus remains unclear.

### K^+^ currents

Patch-clamp experiments and drug studies have indicated the presence of various K^+^ currents in both cultured and freshly isolated zebrafish myocytes.

#### Rapid component of the delayed rectifier K^+^ current (I_Kr_)

I_Kr_ has been observed in both cultured embryonic (Baker et al., 1997) and adult freshly isolated myocytes from zebrafish (Nemtsas et al., 2010). In adult hearts, the I_Kr_ blocker E4031 prolonged zebrafish atrial and ventricular APs (Nemtsas et al., 2010) and QTc interval in a dose-dependent manner (Tsai et al., 2011). These observations are in agreement with findings in humans (Jost et al., 2005). In addition, E4031 decreased heart rate, suggesting a role for I_Kr_ in zebrafish pacemaker formation (Tsai et al., 2011), consistent with findings in mammalian studies (Ono and Ito, 1995).

Scholz et al. (2009) analyzed in Xenopus oocytes the biophysical properties of heterologously expressed cloned zebrafish orthologue (*zERG*) of the human ether-à-go-go-related gene *hERG*, encoding the pore-forming subunit of I_Kr_. *zERG* conduct rapidly activating and inactivating K^+^ currents. However, compared to *hERG*, the half-maximal activation voltage of *zERG* is shifted toward more positive potentials and the half-maximal steady-state inactivation voltage is shifted toward more negative potentials. *zERG* activation is slowed while deactivation is accelerated significantly, but the time course of *zERG* during AP clamp experiments is highly similar to that of *hERG*. Therefore, the authors concluded that zebrafish represent a valuable model to investigate human I_Kr_ channel-related diseases (Scholz et al., 2009). Indeed, a number of studies have applied zebrafish for investigations into human syndromes associated with both loss and gain of I_Kr_ (see below).

#### Slow component of the delayed rectifier K^+^ current (I_Ks_)

In their voltage clamp experiments on isolated adult myocytes, Nemtsas et al. (2010) observed no effect of the I_Ks_ blocker HMR1556 on membrane currents, suggesting that I_Ks_ is absent in zebrafish myocytes. During AP measurements in intact zebrafish hearts, they observed an unexpected prolongation of AP duration by HMR1556 due to a reduction in I_Ca,T_ (Nemtsas et al., 2010). In contrast, Tsai et al. (2011) mentioned expression of the *KCNQ1* transcript (which underlies part of I_Ks_) in zebrafish myocardium, and found that the I_Ks_ blocker chromanol 293B prolonged both QTc interval and AP duration in a dose-dependent manner in isolated adult zebrafish hearts. In addition, chromanol 293B was also able to decrease heart rate in this study (Tsai et al., 2011). In human ventricular myocytes, I_Ks_ is present but I_Ks_ blockade only results in significant AP prolongation when the “repolarization reserve” is attenuated or under conditions of sympathetic activation (Jost et al., 2005). Clearly, further studies are needed to elucidate the contrasting findings of I_Ks_ blockade on the zebrafish AP.

#### Ultrarapid component of the delayed rectifier K^+^ current (I_Kur_)

Baker et al. (1997) mentioned as “unpublished work” the presence of a K^+^ current with the properties of I_Kur_ in cultured embryonic zebrafish myocytes. However, no other studies have as yet provided evidence of I_Kur_ in zebrafish myocytes. I_Kur_ and I_Kur_-related channel proteins are absent/small in human ventricular myocytes, but is the major repolarizing current in atrial myocytes (Amos et al., 1996; Wettwer et al., 2004; Ordög et al., 2006). Additional studies are thus required to address the presence and function of I_Kur_ in zebrafish myocytes.

#### Inward rectifier K^+^ current (I_K1_)

Nemtsas et al. (2010) measured I_K1_ during depolarizing ramp pulses as Ba^2+^ sensitive current. I_K1_ was observed in both isolated adult atrial and ventricular myocytes, but I_K1_ was ≈5 times larger in ventricular myocytes. These observations are consistent with findings in mammals (Koumi et al., 1995; Amos et al., 1996; Panama et al., 2007). I_K1_ regulates the late phase of AP repolarization and stabilizes the resting membrane potential, thus atria of zebrafish hearts may be more susceptible to diastolic depolarization compared to ventricle. Indeed, diastolic depolarization is observed in atria, but not in the ventricle, of intact zebrafish embryonic hearts (Jou et al., 2010).

#### Transient outward K^+^ current (I_to1_)

Again, the presence of a K^+^ current with the properties of I_to1_ in cultured embryonic zebrafish myocytes has so far only been mentioned as “unpublished work” by Baker et al. (1997), and no other studies have provided evidence of I_to1_ in zebrafish myocytes. Since I_to1_ is a prominent current determining human atrial and ventricular AP morphology (Shibata et al., 1989; Amos et al., 1996), further studies are essential to investigate the presence and function of this current in zebrafish heart.

#### Acetylcholine-activated K^+^ current (I_K,ACh_)

In intact adult zebrafish hearts, atrial but not ventricular APs are abbreviated upon exposure to carbachol, an agonist for I_K,ACh_ (Nemtsas et al., 2010). Thus, atrial myocytes of zebrafish display functional I_K,ACh_ in agreement with findings in human (Dobrev et al., 2001). In human, acetylcholine activates I_K,ACh_in both atrial and ventricular myocytes, with however a three-times smaller current and a greater half-maximal stimulation concentration in atrial myocytes (Koumi and Wasserstrom, 1994). Additional detailed studies in zebrafish are required to excluded the presence of I_K,ACh_ in zebrafish ventricular myocytes.

### Hyperpolarization-activated “funny” current

Patch-clamp analysis of cultured myocytes from zebrafish embryos (Baker et al., 1997) and isolated adult myocytes (Warren et al., 2001) reveals the prominent presence of the hyperpolarization-activated “funny” current (I_f_), also known as the pacemaker current (I_h_). In adult zebrafish myocytes, I_f_ has chamber-specific properties, i.e., the atrial I_f_ density is larger than the ventricular I_f_ density (Warren et al., 2001). The clear presence of I_f_ in atrial and ventricular zebrafish myocytes contrasts with findings in human, where I_f_ is mainly found in cells of the conduction system (Han et al., 2002; Verkerk et al., 2007), is small in atrial myocytes (Hoppe and Beuckelmann, 1998), and only observed in ventricular myocytes during pathophysiological conditions such as heart failure (Cerbai et al., 2001). Nevertheless, a functional role for I_f_ in sinoatrial pacemaking and heart rate regulation has also been described in zebrafish (see below; Baker et al., 1997; Warren et al., 2001).

### Na^+^-Ca^2+^ exchange current (I_*NCX*_)

Patch-clamp analysis of the Na^+^-Ca^2+^ exchange current (I_NCX_) has not yet been performed in zebrafish myocytes. However, Langenbacher et al. (2005) have reported the presence of a *NCX1* zebrafish homologue (i.e., *NCX1h*) in the heart of zebrafish, and knockdown studies indicate a functional role for NCX1 in maintaining normal Ca^2+^ homeostasis in the zebrafish heart, as discussed below.

## Excitation-contraction coupling

The zebrafish heart displays clearly visible contractions and these are frequently used to assess the beating rate of the whole heart. Isolated myocytes of adult zebrafish show clear cross-striations, indicating the presence of sarcomeres (Brette et al., 2008; Nemtsas et al., 2010), but zebrafish ventricular myocytes lack t-tubules (Brette et al., 2008). The latter observation contrasts with ventricular myocytes from mammals. It is thought that t-tubules allow the excitation wave to spread from the cell surface deep into the muscle fibers for efficient release of Ca^2+^ from the sarcoplasmic reticulum (SR) (for review, see Brette and Orchard, 2003). The lack of t-tubules thus may have consequences for Ca^2+^ transients, but it is also possible that due to the small diameter of the zebrafish myocytes, t-tubules are not required.

Experimentally, Ca^2+^ transients can be visualized and measured in both single adult myocytes (Zhang et al., 2011) and whole embryonic hearts (Jou et al., 2010). Figure 1C shows a typical example of a Ca^2+^ transient recorded in an isolated ventricular myocyte (Figure adapted from (Zhang et al., 2011)). In the intact embryonic heart, Ca^2+^ transients are shorter in atria then in ventricle, and this correlates with the time course of atrial and ventricular APs (Jou et al., 2010; Nemtsas et al., 2010; Wythe et al., 2011). The exact pattern and mechanisms of excitation-contraction coupling in zebrafish myocytes has not yet been studied in detail. In the mammalian myocardium, Ca^2+^ released from the SR is the main source for generating Ca^2+^ transients. Compared to mammals, however, the SR in lower vertebrates is underdeveloped, has a lower ability to store and release Ca^2+^, and has less importance in excitation-contraction coupling [see Xie et al. (2008), and primary refs cited therein]. To assess the role of SR function in generating Ca^2+^ transients in zebrafish myocytes, Xie and colleagues (2008) recorded Ca^2+^ transients before and after the addition of caffeine in embryonic hearts. The caffeine-induced depletion of SR Ca^2+^ increased diastolic Ca^2+^ levels, as well as Ca^2+^ transient amplitudes. However, caffeine did not halt the repetitive Ca^2+^ transients, implying that Ca^2+^ entry across the sarcolemmal membrane is sufficient for a relatively synchronous and uniform rise in whole cell intracellular Ca^2+^ concentration. These data thus indicate the presence of a functional SR in zebrafish myocytes, although the effects of caffeine were relatively modest. More recently, Zhang and co-workers (Zhang et al., 2011) performed simultaneous recordings of I_Ca,L_, intracellular Ca^2+^, and/or measurements of cell shortening in adult zebrafish myocytes. Their findings suggest that I_Ca,L_ is the major contributor to the activation of contraction at membrane voltages below 10 mV, whereas the contribution of reversed Na^+^-Ca^2+^ exchange becomes increasingly more important at membrane potentials above 10 mV. Crucially, the apparent lesser importance of the SR for excitation-contraction coupling may also make zebrafish myocytes less susceptible to the occurrence of Ca^2+^ aftertransients and subsequent delayed afterdepolarizations. Indeed, morpholino-mediated knockdown of *Serca2*-activity caused embryonic lethality in zebrafish embryos due to defects in cardiac contractility and morphology, but no arrhythmias were observed (Ebert et al., 2005). Thus, zebrafish may not be ideal for investigations into Ca^2+^-modulated arrhythmias, including catecholaminergic polymorphic ventricular tachycardias.

## Zebrafish models of cardiac (patho)electrophysiology

Several studies have investigated the functional effects of altered gene expression of ion channel genes in zebrafish hearts. These mutations were either spontaneously occurring or were identified from large mutagenesis screens or generated through targeted specific knockdown of the gene in question.

### Na^+^ channels and cardiac development

Antisense morpholino knockdown of either of the two cardiac Na^+^ channel orthologs identified in zebrafish (*scn5Laa* and *scn5Lab*) has been shown to severely disrupt early cardiac development in zebrafish (Chopra et al., 2010). Interestingly, pharmacological Na^+^ current blockade did not affect cardiac morphology, suggesting a possible structural role for Na^+^ channels in heart development (Chopra et al., 2010). Similarly, knockdown of brain-type Na^+^ channel ∝- and ß-subunits affects nervous system and motoneuron development in embryonic zebrafish (Pineda et al., 2006; Fein et al., 2008).

### ERG or KCNH2 related models

Langheinrich and co-workers studied *zERG* encoding the pore-forming subunit of I_Kr_ in zebrafish (Langheinrich et al., 2003). Morpholino antisense oligonucleotides targeting *zERG* as well as pharmacological inhibition of *zERG* both elicited dose-dependent bradycardia and arrhythmia in zebrafish embryos, including atrioventricular 2:1 block. Moreover, they identified a mutation in a regulatory domain of the *zERG* channel in the previously identified *breakdance* mutant (*bre*), which is also characterized by a 2:1 atrioventricular block (Chen et al., 1996; Langheinrich et al., 2003). The authors concluded that zebrafish are useful for studying *ERG* function and modulation, and may also be suitable for testing potential QT prolongating effect of drugs. Similary, Arnaout et al. (2007) investigated two recessive *Kcnh2* zebrafish mutants identified with ventricular asystole. Both *Kcnh2* mutations encoded non-functional I_Kr_ channels, and *Kcnh2* mutant zebrafish embryos displayed ventricular AP prolongation, QT interval prolongation, and increased sensitivity to QT prolonging drugs, thus constituting a potential research model for human long QT syndrome (Arnaout et al., 2007). In contrast, the *reggae* mutation (*reg*) was found to reside within the voltage sensor of *zERG* and caused a gain-of-function of I_Kr_ due to defective channel inactivation (Hassel et al., 2008). Accordingly, *reg* mutant adult zebrafish displayed shortened QT intervals, and this mutation has been proposed as a relevant research model for human short QT syndrome type 1 (SQT1; Hassel et al., 2008).

### L-type ca^2+^ channels and cardiac proliferation

One of the first zebrafish mutants displaying a clear cardiac phenotype was *island beat* (*isl*), which presented with a silent and non-contractile ventricle and an asynchronously beating atrium resembling atrial fibrillation (Rottbauer et al., 2001). The *Isl* locus was subsequently found to encode the cardiac L-type Ca^2+^ channel. Interestingly, the atrium of *Isl* mutants was structurally normal, but the ventricle was small and contained relatively few cardiomyocytes. These findings indicate a possible uncharacterized role for L-type Ca^2+^ channels in cardiac proliferation (Rottbauer et al., 2001).

### The *slow mo* gene

A spontaneous mutation in this gene was identified in a particular strain of zebrafish, and it was found that adult zebrafish with a homozygous mutation in the *slow mo* gene were bradycardic (Baker et al., 1997). Through patch-clamp analysis, it was revealed that most cardiac ion currents, including Na^+^, K^+^, and Ca^2+^ currents were unaffected by the recessive *slow mo* mutation. In contrast, it became apparent that the I_f_ was defective (Baker et al., 1997; Warren et al., 2001). Although the exact underlying genetic defect remains unknown, these studies in *slow mo* mutants clearly indicate a functional role for I_f_ in zebrafish sinoatrial pacemaker formation.

### Na^+^-Ca^2+^ exchanger and rhythmicity

Langenbacher et al. (2005) reported the presence of a *NCX1* homologue (*NCX1h*) in the atrial and ventricular myocardium of zebrafish. Using morpholino knockdown assay and positional cloning of the zebrafish *tremblor* (*tre*) locus, the authors demonstrated that defective NCX1h activity results in chaotic cardiac movements and dys-synchronized cardiac contractions due to abnormal Ca^2+^ transients (Langenbacher et al., 2005). Another study on *tre* mutants demonstrated dysregulation of atrial rhythmicity (including fibrillation), a silent ventricle, and severe disruptions in sarcomere assembly (Ebert et al., 2005). Results from these studies thus indicate that NCX1h is required for normal development and rhythmicity in the zebrafish heart.

## Conclusions

Zebrafish embryonic and adult heart rates as well as AP and ECG morphology closely resemble those of humans. However, whether the zebrafish is truly an attractive alternative model for human cardiac electrophysiology depends on the presence and gating properties of the various ion channels in the zebrafish heart. The rapid component of the delayed rectifier potassium current (I_Kr_) remains the best characterized and validated ion current in zebrafish myocytes, and zebrafish may represent a valuable model to investigate human I_Kr_ channel-related disease, including long and short QT syndromes. Arguments against the use of zebrafish as model for human cardiac (patho)electrophysiology include its cold-bloodedness and two-chamber heart morphology, absence of t-tubuli, limited SR function, presence of I_Ca,T_ and I_f_, absence of I_to1_ and I_Kur_, and lowI_Na_ density. Based on the currently available literature, we propose that the zebrafish may constitute a relevant research model for investigating ion channel disorders associated with abnormal repolarization, but may be less suitable for studying depolarization disorders or Ca^2+^-modulated arrhythmias.

### Conflict of interest statement

The authors declare that the research was conducted in the absence of any commercial or financial relationships that could be construed as a potential conflict of interest.
